# Arthroscopic treatment of synovial chondromatosis of hip joint

**DOI:** 10.1186/s13018-020-01928-8

**Published:** 2020-09-11

**Authors:** Yufeng Liu, Ji Li, Ning Ma, Mingyang An, Feng Gao, Bo Hu, Boqing Zhang, Zhigang Wang, Yujie Liu, Chunbao Li

**Affiliations:** 1 Department of Sports Medicine, Chinese PLA General Hospital, Beijing, 100853 China; 2 Institute of Orthopedic, Chinese PLA General Hospital, Beijing, 100853 China; 3 Department of Sports Injury and Arthroscopy Surgery, National Institute of Sports Medicine, Beijing, 100061 China; 4 The Second Department of Orthopedics, Beijing Chaoyang Integrative Medicine Emergency Medical Center, Beijing, 100023 China

**Keywords:** Hip arthroscopy, Synovial chondromatosis, Loose body removal, Hip joint

## Abstract

**Purpose:**

This retrospective study summarized the clinical, radiographic, and arthroscopic manifestation of synovial chondromatosis (SC) of the hip, along with the post-operative effect to discuss the curative effect of arthroscopic management of hip SC.

**Methods:**

Twenty-one patients who underwent arthroscopic surgery from the same surgeon for hip SC were followed up for an average of 45 months. T-shaped capsulotomy was routinely performed in each case. Visual analog scale, range of motion, modified Harris Hip Score, and International Hip Outcome Tool score were collected preoperatively and at the time of the latest follow-up. All patients’ demographics, radiographs, and arthroscopic images were collected to summarize and conclude the similarities and differences of their manifestation.

**Results:**

Large wedged clumps of loose bodies demonstrated distinguishable radiographic, arthroscopic appearance and demanded a different surgical strategy. Postoperative scores were all significantly improved. One case of residual pain and two cases of residual loose bodies with no symptom related were reported at the final follow-up. All but one patient were satisfied with the outcome.

**Conclusion:**

Arthroscopy treatment of hip SC with T-shaped capsulotomy has demonstrated a good result in terms of clinical outcome score, recurrence rate, and complication rate. On the basis of this study, we concluded the clinical performance of large wedged clumps of loose bodies of hip SC.

## Introduction

Synovial chondromatosis (SC) is a rare disease with cartilaginous metaplasia which occurs within the synovium of the joint [1, 2]. It commonly results in the growth of cartilaginous nodules in the synovium, many of which may detach and become loose bodies in the joint and subsequently cause damage to the articular surface and cause osteoarthritis [3]. In the majority of cases, the condition is monoarticular, with hip as the second most frequently affected joint commonly seen on clinical practice [4, 5]. Milgram [3] had defined it as a condition with greater than four osseocartilaginous loose bodies associated with synovial metaplasia.

Early diagnosis of hip SC often means a better prognosis. However, due to the nonspecific and insidious symptom of the disease and regularly false-negative imaging in early stages, the period to confirm definitive diagnosis often exceeds more than 2 years. Complaints usually include nonspecific pain as the main cause for medical consultation, motion limitation (51 to 100%), limp (9.9 to 90%), and locking episodes (19 to 63%) [6, 7], all of which are commonly seen in various hip conditions. Plain radiograph was considered first-line imaging, due to its easy practicability, sensitivity for the most common hip diseases, and common availability, and showed a mean sensitivity of 28 to 83% [6]. CT and MRI could help confirm the diagnosis with a higher accuracy but more costs. Delaying of treatment could cause complications such as joint deterioration and secondary osteoarthritis (OA), finally leading to arthroplasty or handicap [8]. Conservative treatment for hip SC has shown little effect. A broad consensus about the necessity for operative removal of intra-articular loose bodies has already been reached. As a relatively mature technique, open arthrotomy has shown relatively good results [6, 9–11], but causes bigger trauma and longer time to recover. Hip arthroscopy could be used as an approach of management, but also a tool for definitive diagnosis. Arthroscopic management for hip SC appears safer with an approximate 1% minor complication rate [12] and a 7.1% recurrence rate [13]. In addition to that, minimally invasive surgery has been proven to shorten the in-hospital stay and postoperative rehabilitation with possible fully weight bearing. However, the complexity of hip’s anatomy structure and profound soft tissue around it makes hip arthroscopy a technically demanding procedure. Considering the prognosis of hip SC largely depends on the complete removal of scattering loose bodies all over the joint, it requires extremely high standard for physician’s surgical capability.

The aim of this study was to report our own experience of arthroscopic management of hip SC in 21 patients in a retrospective way. T-shaped capsulotomy and synovectomy was performed in all cases. Retrospective analysis of all patients’ demographics, radiographs, and arthroscopic images was performed. Reviewing this gave us a clearer look at the typical risk factors, manifestation, and therapeutic effect of this manner.

## Materials and methods

### Patients

From March 2013 to January 2019, 385 patients had undergone hip arthroscopic surgery and 35 were diagnosed with hip SC according to radiographic, arthroscopic, and pathological manifestation. All 35 patients consented to participate in the study. Demographic data, history, physical examination findings, imaging results, and intraoperative findings were provided. All data were collected prospectively in a computer database following approval by the research ethics board. All operations were performed by the same surgeon (the 2nd corresponding author). Exclusion criteria include the following: (1) history of surgery of the affected hip, (2) grade IV arthritis (Kellgren-Lawrence classification system) under plain radiograph, (3) combined dysfunction of other joint, (4) history of joint infection, (5) history of autoimmune disease, (6) history of chronic consumptions such as tumor and tuberculosis, and (7) less than 2 years of follow-up.

### Clinical and radiographic evaluation

The study was conducted through retrospective examinations of all the patients. We inquired different symptoms from every patient, including pain, locking episode, motion limitation, and limping. FADDIR test and FABER test were conducted in addition to normal physical examination. Visual analog scale (VAS), range of motion (ROM), modified Harris Hip Scores (mHHS), and International Hip Outcome Tool (iHOT12) were measured and inquired before surgery and at final follow-up.

Plain radiographs of anteroposterior views of pelvic with the affected hip joint were taken. Kellgren-Lawrence classification system was used to assess the grade of OA of hip joint. Radiographic evidence for diagnosis of SC was mainly detection of loose bodies. Medial joint space widening could also be an indicator for reasonable speculation. Other issues which required attention include FAI, osteophytes, subchondral cysts or sclerosis, or bone erosion. In consideration of non-ossified loose bodies getting in the way of a confirmative diagnosis, MRI was also performed on all patients preoperatively. Labral tears [4] were detected in MRI, which was also a condition need to be dealt with. Extra attention was paid to the size and location of loose bodies, thickening synovium, and presence of joint effusion for surgery planning.

### Surgical technique

After general anesthesia, the patient was positioned supine in the traction bed with marker made on the skin. The area of perineum was carefully protected. Standard skin preparation and draping was finished. Traction of ipsilateral lower limbs was performed to confirm at least 8–10 mm of joint-space access, with the hip abducted 10° to 15° and internal rotated. An anterolateral approach (ALA) was firstly taken under fluoroscopic control approximately 1 cm superior and anterior to the anterior edge of the greater trochanter, and then an anteromedial approach (AMA) was established under arthroscopic surveillance. Capsulotomy was performed between two portals. The central compartment was investigated first. Standard surveillance of the acetabulum, labrum, cartilage of femoral head and acetabular fossa, and round ligament was performed. Loose bodies and hyperplasia and hyperemia of synovium were addressed emphatically. Smaller loose bodies were rinsed out with cannula and larger ones were clamped out. Curette, curved scraper, and slide winder blade were used to wipe out the synovial tissue. Cartilage lesions were evaluated according to Outerbridge classification, and then a chondroplasty or microfracture was performed accordingly. Damaged and degraded round ligament was trimmed. Overcoverage of acetabulum (Pincer-type dysplasia) has restored normal anatomy by abrasion of hyperplastic bone. Before management of labral pathology, distal anterolateral approach (DALA) was created to facilitate suture and anchor placement to ensure safety. T-shaped capsulotomy was routinely performed along the fiber direction of the iliofemoral ligament to connect the three approaches created. Labrectomy, trimming, or stitches with suture anchor were performed according to the quality and level of damage of the labrum. Peripheral compartment was accessed after central compartment was addressed. Traction was relaxed, and the hip joint was bended at the range of 35~45° to restore relaxation state of the articular capsule. Extra attention was paid to explore the anteromedial and posterolateral recess to avoid residue loose bodies and synovium. Cam-type FAI was addressed with abrasion of osteophytosis. Dynamic inspection under arthroscopic surveillance was implemented to ensure no impingement between the acetabular bone and femoral neck during internal and external rotation. Synovectomy was performed thoroughly. Capsule was closed. The number of loose bodies was counted after the operation. Big clumps of loose bodies formed by several small ones were counted as one.

### Rehabilitation

Touchdown weight bearing with crutches was allowed at the 1st day after operation. Passive range of motion was restricted up to 90°. In the first 4 weeks, weight bearing as tolerated with crutches was allowed until full weight bearing was reached. Active range of motion could be gradually increased up to 90° without external rotation or extension from resting position. Isometric contraction of muscles of the lower extremities was encouraged. At fifth to twelfth week, full weight bearing and normal range of motion were achieved. Daily activity could be restored with or without crutches as needed. Low impact sports as tolerated were encouraged. High-impact sports were not recommended until 6 months.

### Statistical analysis

The SPSS 17.0 (SPSS Inc., Chicago, Illinois) was used to analyze all the data collected. Normal distribution of every data was checked before other analyses. The value of every affected factor, the VAS, ROM, mHHS, and iHOT12 were calculated and analyzed to tell the significance using paired t tests with *P* < 0.05 considered statistically significant.

## Results

### Clinical presentation and postoperative outcome

**Fig. 1:**
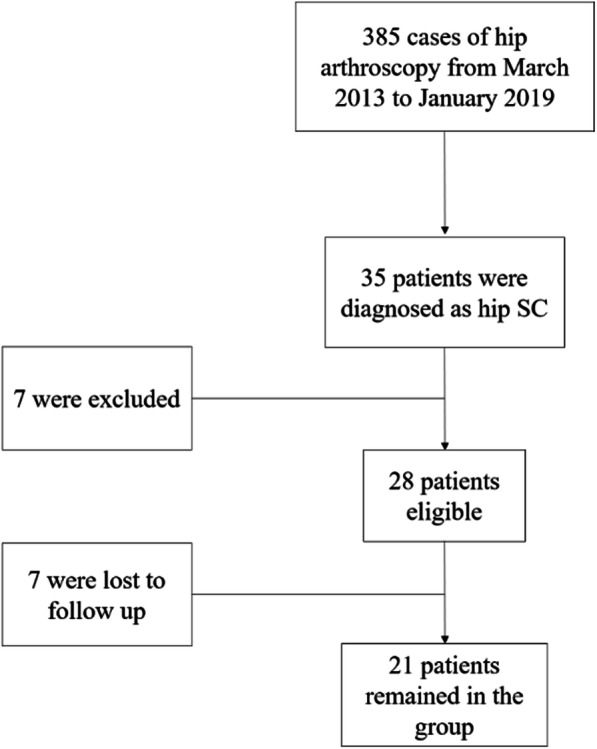
Flowchart of the enrollment of patients. Twenty-one patients included into the final group

Twenty-one patients were included into the final group (Fig. 1) with an average follow-up of 45 months (range, 24–69 months). All 21 cases complained symptoms of ipsilateral hip pain, 20 of limited mobility, along with seven cases of locking episodes. Most commonly used terms to describe pain was swelling and dull, some of which accompanied with radiating pain of the ipsilateral thigh and knee. In the 21 patients, 20 (95.2%) were satisfied with the postoperative outcome. No complaining of pain was presented at the final follow-up except for 1 case. Range of motion was restored back to normal in 20 patients. Locking was gone in all patients. Limping was spotted in 19 out of 21 cases preoperatively, and all gained normal gait at the final follow-up except for one. Atrophy of gluteus medius muscle came up in 17 cases before operation, and only three remained at the final follow-up. FADDIR and FABER tested positive in 14 and 13 cases and were reduced to three and four cases respectively. ***VAS,*** ROM, mHHS, and iHOT12 score all improved significantly after the procedure (*P* < 0.001) (Table 1). There was one complication of continuous pain occurred. Ipsilateral hip pain still existed with limited mobility and limping. Atrophy of the gluteus medius muscle of the affected hip still existed after surgery, and there was little improvement of VAS, ROM, mHHS, and iHOT12 score. We contribute this to the late phase development and severe damage of cartilage (Outerbridge stage IV). Progression of arthritis was shown. This patient eventually ended up with total hip replacement. Two cases of residual loose bodies were identified on postoperative plain radiograph without associated symptom, and patients were satisfied with the outcome with no further consultation so far. No other common complications such as neurovascular injury, deep vein thrombosis, femoral head necrosis, and infection took place.

**Table 1:** Comparison of clinical outcomes and range of motion

	Pre-op	Post-op	*P* value^‡^
VAS score	5.90 ± 0.89	2.24 ± 1.14	0.0000***
ROM	153.57 ± 26.41	245.33 ± 36.17	0.0000***
mHHS	36.43 ± 7.05	85.87 ± 18.12	0.0000***
iHOT12 score	69.67 ± 12.65	96.57 ± 17.56	0.0000***

Data were expressed as mean ± SD (range) unless otherwise indicated

*SD* standard deviation

*P < 0.05; **P < 0.01; ***P < 0.001

^‡^Unpaired *t* test. *P* < 0.05 was defined as significant

### Pre-operative radiographic findings

**Fig. 2:**
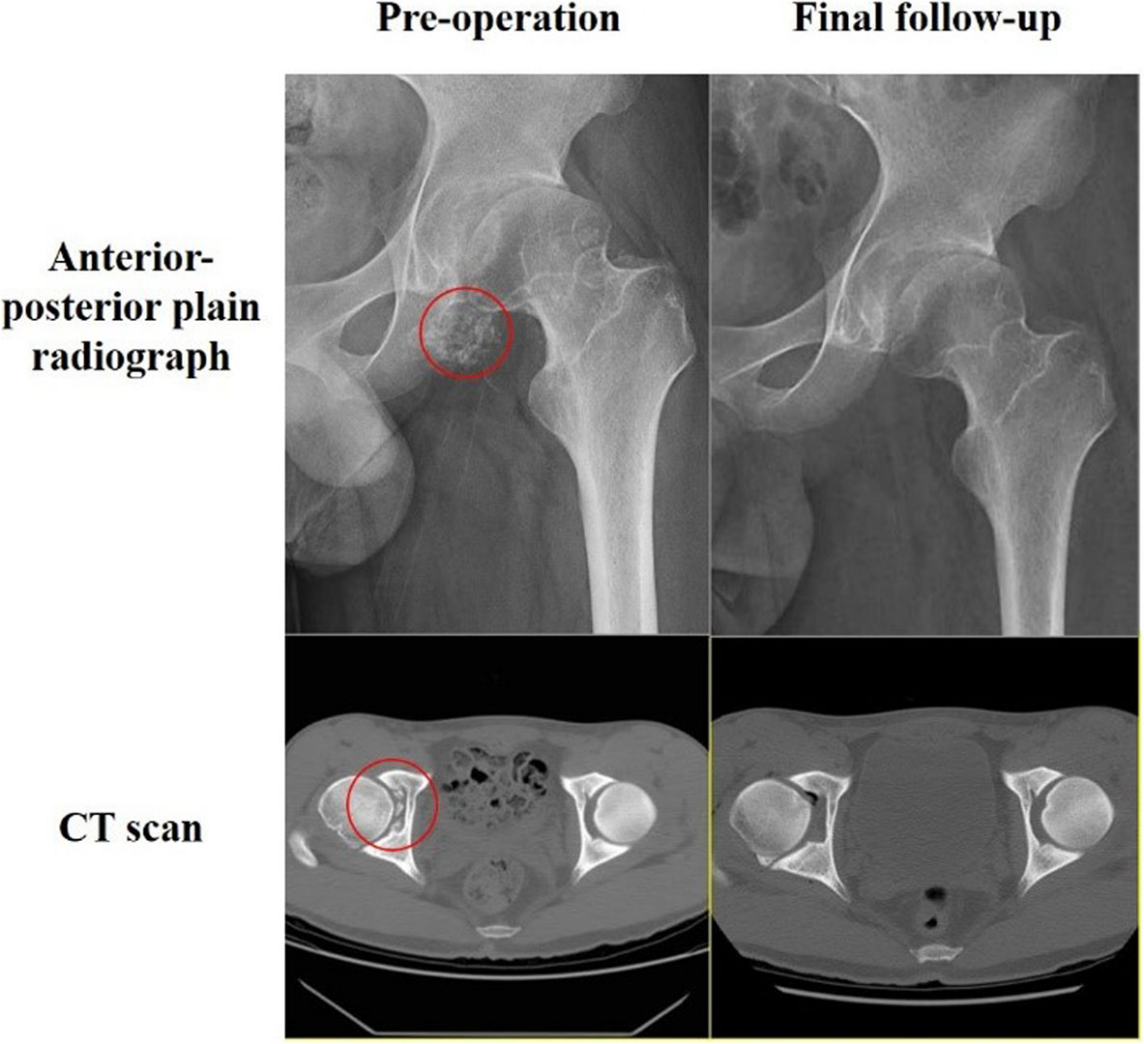
Anterior-posterior plain radiograph and CT scan at pre-operation and the final follow-up. Red circles indicate losse bodies

Most cases (20 cases) presented with osteoarthritis of the affected hip in plain radiograph pre-operatively (Table 2), and all showed improvement in the post-operative plain radiograph at the final follow-up except for one case. Loose bodies could be identified in most cases (18 cases) on plain radiograph. The most typical presentation manifested as cloudy shadow around hip joint + with distinct ossified borders of each individual intra-articular radiopaque loose bodies (Fig. 2). Additional CT was needed in a rare condition (Fig. 2). Three cases of atypical radiological appearance of loose bodies with one large and unified fragment with ossified border detached from the acetabulum were identified (Fig. 5a). The fragment shares the same concavity with the acetabulum, making it easy to be dismissed. CT and MRI performance were all in consistency with radiograph appearance (Fig. 5b, c). Two cases with negative plain graphs were confirmed by MRI. Cam- and Pincer-type FAI were identified on the plain radiograph with bony hyperplasia at the junction of femoral head and neck and overcoverage of the acetabulum. All cases presented with synovial thickening and joint effusion on preoperative MRI. Labrum abnormality was presented in all cases, with 18 tear and 3 degeneration, detected on MRI. One patient with unspecific symptoms demonstrated both negative plain graph and MRI until non-ossified loose bodies were seen under arthroscopy. MRI was not performed as a routine postoperatively. At the final follow-up, most cases were absent of loose bodies (Fig. 2) except for two.

**Table 2:** Pre-operative radiographic findings

		No. of hips (%)	
		Plain radiograph	MRI
Osteoarthritis	Stage 0	1 (4.8)	1 (4.8)
	*Stage 1*	11 (52.4)	11 (52.4)
	Stage 2	7 (33.3)	7 (33.3)
	Stage 3	2 (9.5)	2 (9.5)
	Stage 4	0	0
Lose bodies		18 (85.7)	20 (95.2)
FAI	Cam	5 (23.8)	5 (23.8)
	Pincer	1 (4.8)	1 (4.8)
	Mixed	14 (66.7)	14 (66.7)
Synovial thickening		0	21 (100)

### Arthroscopic findings

**Fig. 3:**
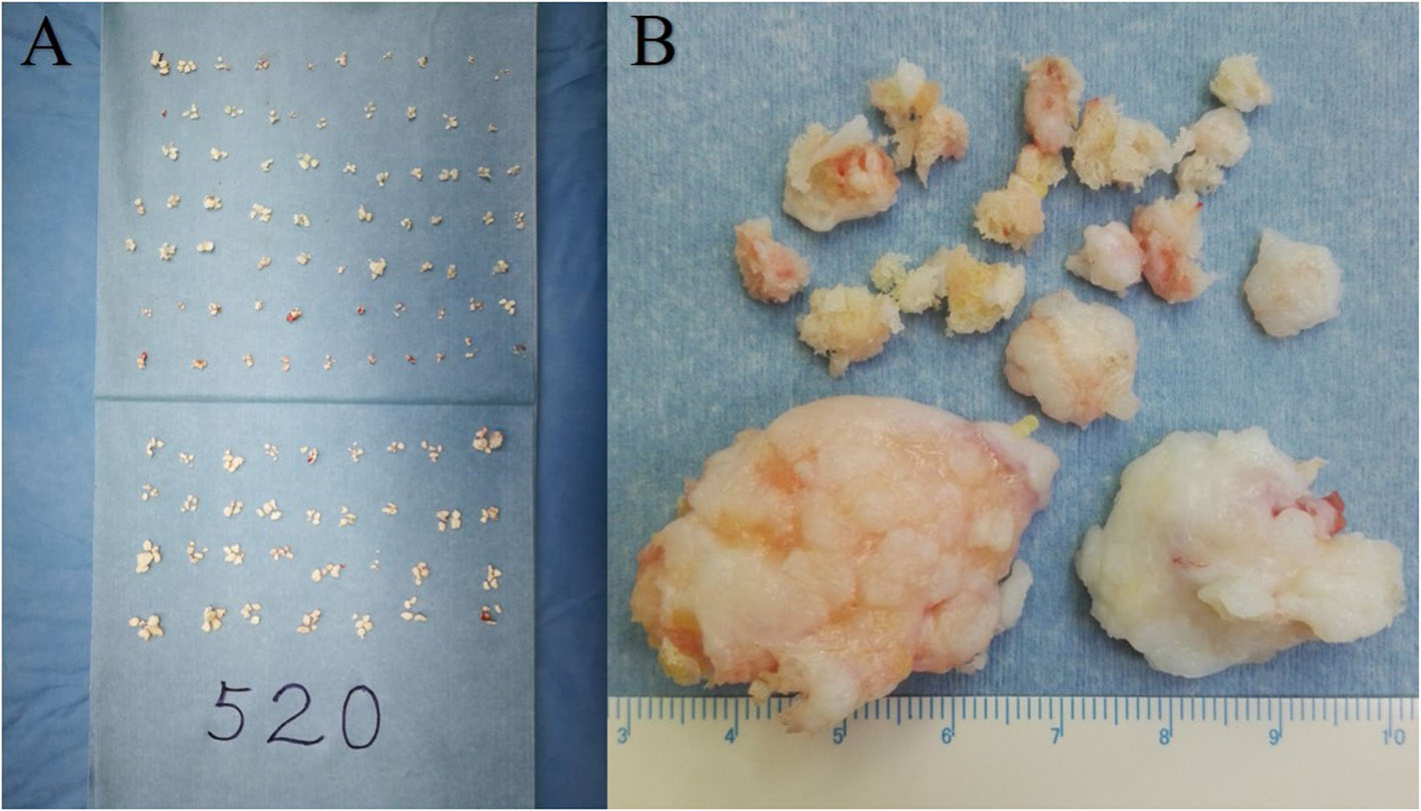
Varies amounts and sizes of loose bodies. **a**: largest amount. **b**: largest size

**Fig. 4:**
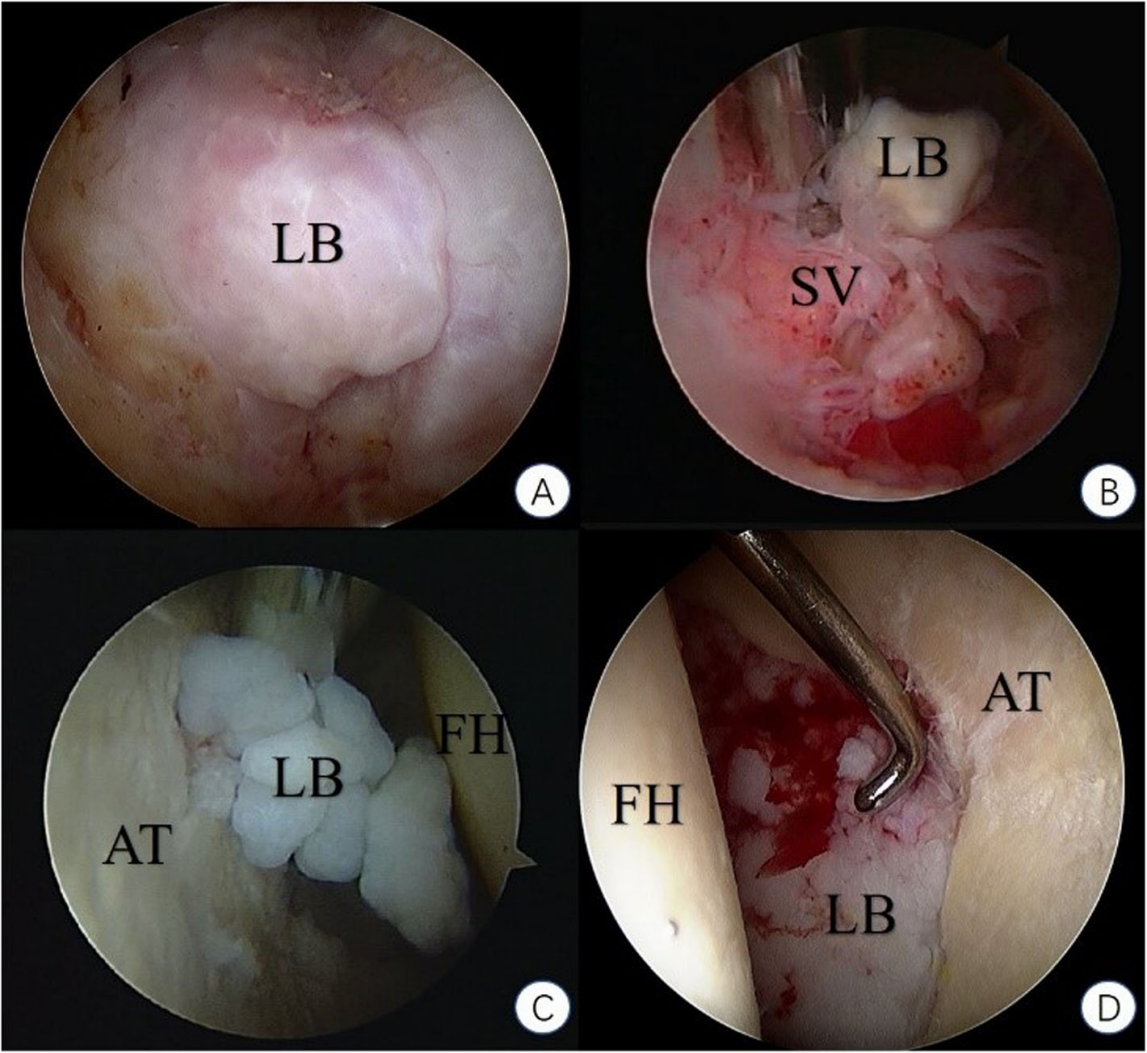
Classification of all the loose bodies according to their arthroscopic appearance on the basis of Milgram’s theory of classification of loose bodies. **a**: Milgram stage Ι. **b**: Milgram stage ΙΙ. **c**: Milgram stage ΙΙΙ. **d**: wedged-clump type. (LB: losse bodies. SV: synovium. FH: femoral head. AT: acetabulum.)

More cases showed acetabular abnormalities than the femoral head. Combined lesions at both ends could be seen in 19 patients. The cartilage of the femoral head was relatively well preserved compared to the acetabular cartilage, with more cases in grade I or II (15 cases) than grade III or IV (4 cases), which was the other way around at the acetabulum (7 cases in grade I or II and 13 cases in grade III or IV). Loose bodies could be seen under arthroscopy in all cases, with a mean number of 89 (ranging 7~520), the diameter of which ranging from 1 to 40 mm (Fig. 3). The morphology of loose bodies varied, from small chips scattered in the articular joint to large clustered aggregation wedged into the acetabulum fossa. We classified all the loose bodies we found according to their arthroscopic appearance on the basis of Milgram’s theory of classification of loose bodies [3] (Fig. 4). In addition to this, we classified the type of large wedged clumps of loose bodies aggregated by multiple small ones separately as a single category (Fig. 4d). This category did not include randomly floating clumps of loose bodies. It referred specifically to the big clumps forged deeply into the acetabulum fossa. The diameter of these loose bodies was relatively bigger, which was the result of aggregation and fusion of piles of small ones. Some of these may broke into small pieces if clamped too hard. Probe was used to dig these loose bodies out of the fossa. We referred this type of loose bodies as the wedged clump type. Concurrent smaller loose bodies could be found in the central and peripheral compartment. All cases showed hypertrophy and hyperemia of the synovium (Table 3). Synovectomy was performed in all cases simultaneously. Labral abnormalities were consistent with MRI findings. Osteophytosis at the junction of head and neck and at the edge of acetabulum could be seen accordant with preoperative plain graph and was addressed accordingly.

**Table 3:** Arthroscopic findings

		No. of hips (%)
Synovial hypertrophy		21 (100)
Labrum	Tear	18 (85.7)
	Fraying and degeneration	3 (14.3)
Osteophytosis	At junction of head and neck	5 (71.4)
	At acetabulum	1 (4.8)
	At 2 ends	14 (66.7)
Acetabulum cartilage defect	Outerbridge stage 0	1 (4.8)
	Outerbridge stage I	3 (14.3)
	Outerbridge stage II	4 (19)
	Outerbridge stage III	6 (28.6)
	Outerbridge stage IV	7 (33.3)
Femoral head cartilage defect	Outerbridge stage 0	2 (9.5)
	Outerbridge stage I	8 (38.1)
	Outerbridge stage II	7 (33.3)
	Outerbridge stage III	3 (14.3)
	Outerbridge stage IV	1 (4.8)

## Discussion

In this study, we performed a retrospective analysis of 21 patients with hip SC who underwent arthroscopy surgery. All but 1 patient have thus far reported a good outcome, with a pain-free joint, functional range of motion, and return to their normal activities of daily living (including sport). Upon review of these cases, certain phenomenon deserved extra attention to be paid to.

Milgram classified loose bodies of SC into three types [3]: type I or early stage, where there are synovial-based masses without intra-articular loose bodies; type II or intermediate stage, where there are synovial-based masses and free intra-articular bodies; and type III or late stage, with multiple free osteochondral loose bodies without demonstrable synovial disease. In our study, six additional wedged clump type were found besides three types described by Milgram (Table 4). They appeared to be aggregation of loose bodies wedged, compressed, and accumulated at the horseshoe fossa, shaping like pomegranate seeds, which makes it difficult to be observed (Fig. 4d). There was no entanglement between the loose bodies and synovium, distinguishing them from Milgram type II or intermediate stage. The authors believe that the unique morphology of wedged clump-type loose bodies were due to the specific enarthrodial anatomy and biomechanical structure. Constant compression from the femoral head wedged numerous small loose bodies into the acetabulum fossa and forged these into a whole big clump. Therefore, as a classification designed for SC of all joints, Milgram’s theory does not classify these as a separate category. Many physicians have described the large clumps of loose bodies formed by smaller ones and lodged into the acetabulum fossa [7, 14–17], proving its universal existence. We believe that this type of loose bodies distinguish themselves in terms of radiographic appearance, arthroscopic morphology, and surgical strategy to deal with. Therefore, we conclude our findings and hope it will provide certain guidance for fellow physicians.

**Table 4:** Types of loose bodies

	Milgram stage I	Milgram stage II	Milgram stage III	Wedged clump type
Male	2 (9.5)	1 (4.8)	4 (19)	4 (19)
Female	1 (4.8)	2 (9.5)	5 (23.8)	2 (9.5)
Total	3 (14.3)	3 (14.3)	9 (42.9)	6 (28.6)

**Fig. 5:**
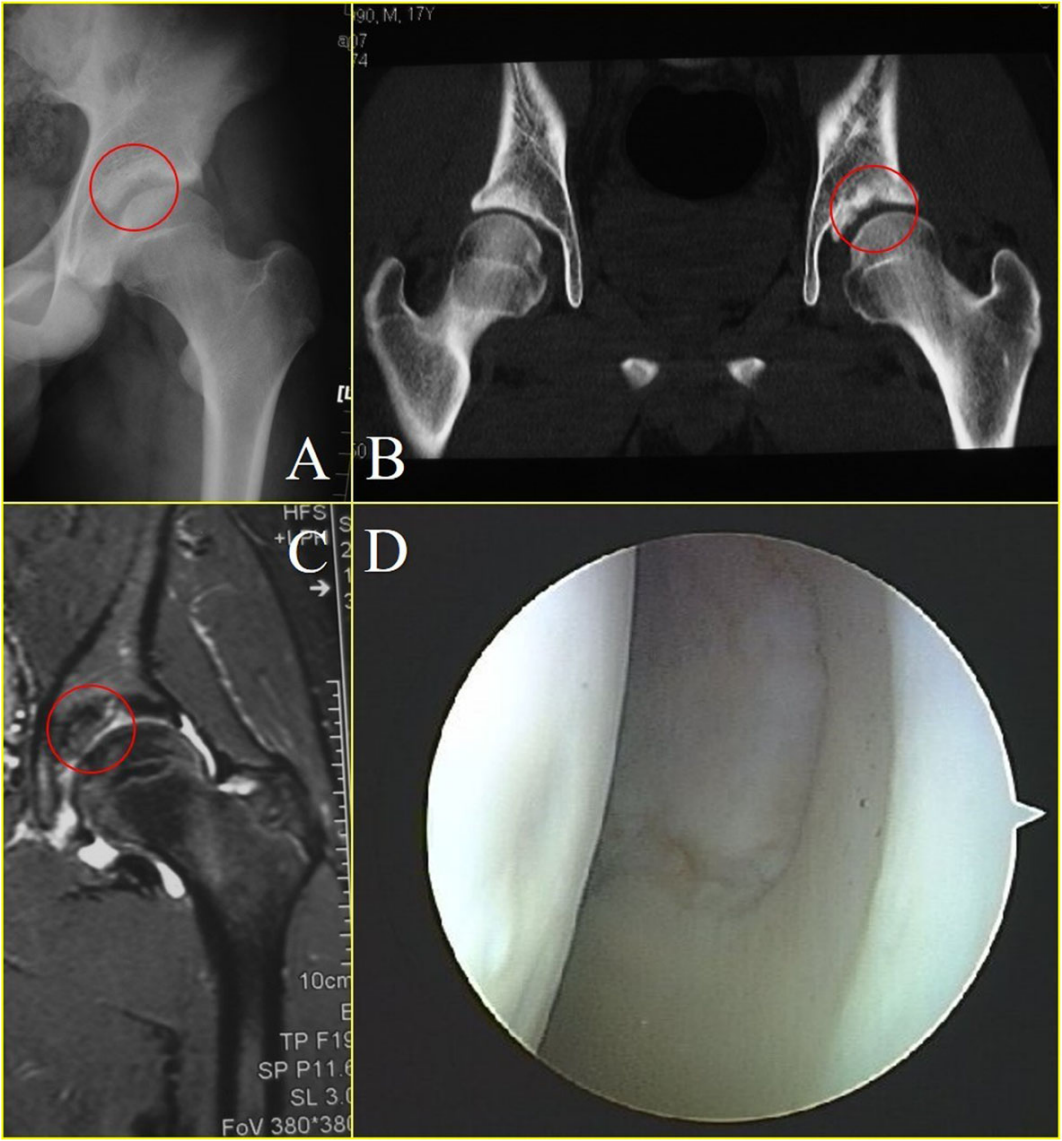
An atypical radiological appearance identified on plain radiograph of three patients with wedged clump type loose bodies. Red circle indicate loose bodies. (**a**: anteroposterior plain radiograph. **b**: CT scan. **c**: MRI. **d**: arthroscopic view.)

SC’s tendency of recurrence and 5% of malignant transformation to synovial chondrosarcoma or chondrosarcoma make it cannot be neglected [18–20]. Due to the non-specific symptoms, the diagnosis of hip SC largely depends on radiographic findings. However, plain radiographs show the presence of loose bodies in only 50% of the cases [21], as 1/5~1/3 of loose bodies were not ossified [22, 23], making it notoriously elusive in imaging studies. In our study, loose bodies were detected in most cases on plain radiograph, with only three cases of negative finding. We conclude the relatively high resolution to the late stage development of most cases, which is corresponded with the arthroscopic findings. Most patients were reluctant to undergo any surgical management without actual evidence of pathological change until the symptoms became intolerable or radiopaque intra-articular loose bodies became ossified and visible on radiograph, which resulted in irreversible damage to the joint and consequently unsatisfying prognosis. MRI has become international gold standard in imaging SC in the pre-mineralization phase. Being a synovial pathology, morphologic changes are demonstrated best by contrast-enhanced T1-weighted fat-suppressed, axial T2-weighted, and coronal T1-weighted sequences [5]. As for the smaller loose body, MRI only presented as hip hydrops [9]. An atypical radiological appearance was identified on plain radiograph of three patients with wedged clump type loose bodies (Fig. 5). It was very similar to the image appearance of OCD (osteochondritis dissecans) with a large piece of ossified fragment dislodged from the acetabulum and fell into the joint space. Both the shape of wedged clump-type loose bodies and OCD fragment were found to be fitting the contour of the acetabulum, making them indistinguishable radiographically. Differential diagnoses between these two conditions were ambiguous, as neither symptoms nor radiological manifestation were specific. Arthroscopic surgery could serve as both treatment and diagnostic tool in this dilemma. In our study, three cases of the six wedged clump type presented with large ossified fragment fitting the concavity of acetabulum. Small amount of independent loose bodies were found in the central and peripheral compartment under arthroscopic inspection. We believe that the relatively smaller quantity of scattered loose bodies was due to the fact that most loose bodies were compressed into the acetabular fossa and forged into one big clump. These additional small ossified loose bodies could be an indication for the diagnosis of SC, but were sometimes invisible on plain radiograph due to their insidious location or non-ossified status.

Different strategies were applied according to different types of loose bodies. Type I (Fig. 4a) was characterized by loose bodies confined in the synovium. At this stage, synovium remains its chondromateplastic potential, so the authors believe that a total synovectomy is essential to avoid recurrence and achieve a better prognosis. Loose bodies should be carefully searched and clamped out before synovectomy. Extra caution needs to be taken as to not break free and lost track of the loose bodies to reside intra-articularly when performing synovectomy. In type II (Fig. 4b), some “loose bodies” were not loosen any more. They were located at and attached to the free side of the synovium, making them generally hard to miss. Loose bodies should be taken care of at first, and then other pathological conditions. In our opinion, synovium should also be completely resected at this stage to prevent postoperative recurrence. In type III (Fig. 4c), scattered loose bodies were not confined in a specific area and moving freely in the joint capsule. The shape of loose body varies from round, oval, rice-shaped to antlers, and so was the size. Flexing, extending, and rotating of the hip joint after relaxation and traction during the operation can effectively avoid the missing of loose bodies. Observation of the lateral part of the hip was more accessible when the hip was fully flexed and external rotated, which is relatively easy compared to the medial part, which could be best inspected with the hip extended and internal rotated. The following methods were taken to avoid blind angle: (1) interchange the approach of arthroscopy and surgical instrument, (2) using arthroscopy of 30° and 70° in turn, and (3) expanding the internal access of the approach to increase the range of the movement of scope. Synovectomy should be restricted to the hypertrophy and hyperemia area. When dealing with the wedged clump type (Fig. 4d), due to the embedding of the loose bodies into the acetabular fossa, it is difficult for the general surgical instruments to reach to. Curved scraper and slide winder blade were needed to remove all the loose bodies out. Some of the wedged big ones were in fact the aggregation of many small loose bodies hinged together, each with intact calcified or cartilaginous surface of its own. Clamping too hard may result in morselization of the aggregation and scatter of the floating loose bodies, increasing the complexity and time duration of the surgery. However, when the wedged loose bodies were too big to maneuver, breaking down of the aggregation might be inevitable. A half pipe cannula might be used to secure the easy passage of the small chipped loose bodies.

**Fig. 6:**
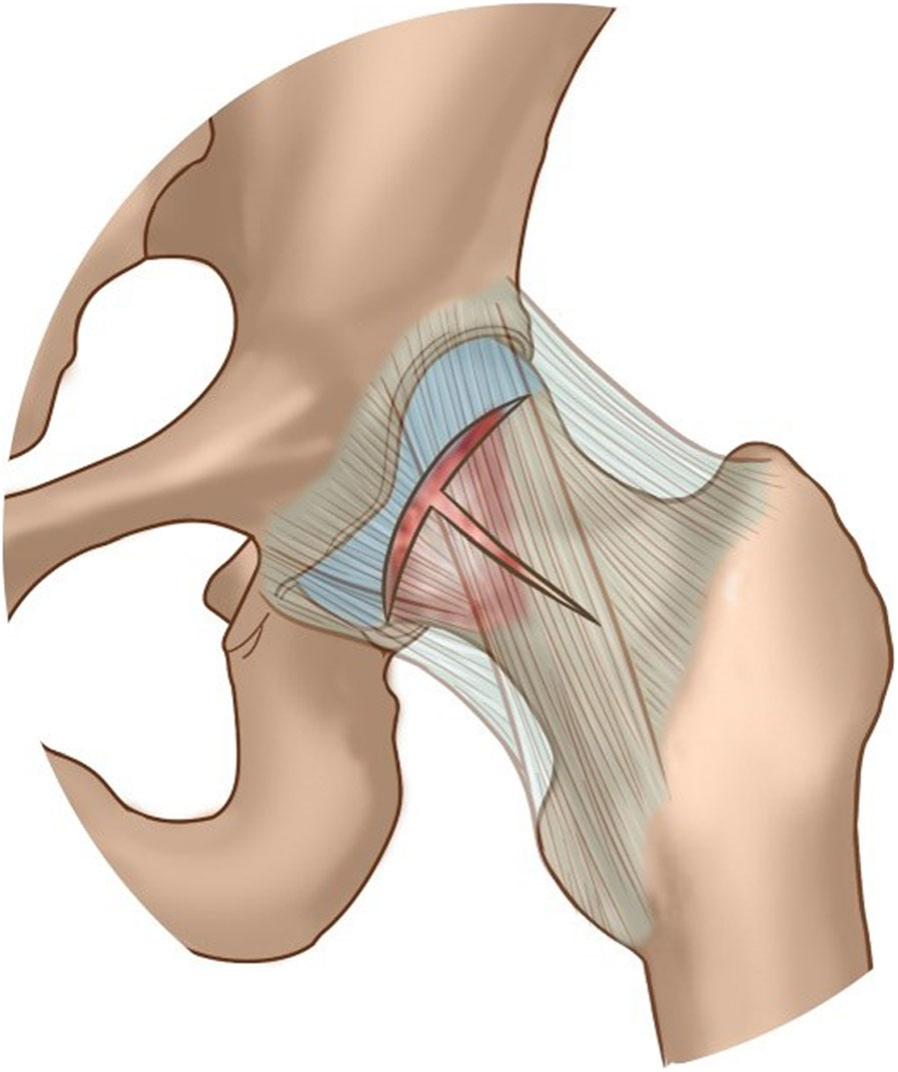
Diagram of T-shaped capsulotomy of hip joint: between the ALA, AMA, and DALA on capsule along the axis of femoral neck, along the iliofemoral ligament, and perpendicular to the intertrochanteric line

The precise congruency of the articulation of acetabulum and femoral head makes the hip an intricate structure. Despite its relatively large size, the intra-articular space of the hip joint for surgical maneuverability is relatively insufficient. T-shaped capsulotomy was first brought up by Horisberger et al. [24] for the management of FAI. It is an incision between the ALA, AMA, and DALA on the capsule along the axis of femoral neck, along the iliofemoral ligament, and perpendicular to the intertrochanteric line (Fig. 6). It aims to increase the exposure for better arthroscopic surveillance of peripheral compartment and space for instrument operability to deal with certain central compartment pathology. In this study, it was carried out in each case to facilitate instrument maneuverability and thorough inspection of capsular recess. It allows the removal of loose bodies as many as possible and does not add substantially to the case time, complications, or complexity. Moreover, a complete exposure becomes more accessible with this cut and therefore result in a more thorough synovectomy without taking the risk of a series of potential complications resulted by dislocation of the hip joint. In addition to that, FAI is known to be commonly associated with hip SC [25, 26], along with labrum abrasion and tear. T-shaped capsulotomy facilitates not only the resection of osteophytes at the junction between the femoral head and neck, but also debridement, stitches, and suture anchor placement to manage the labral pathology.

It should be noted that the following problems still exist in this study: limited number of cases were included in this study, and with a larger size of samples, the credibility will be more authoritative. Longer follow-up time is also needed for better credibility. We believe that our experience and operative guidance followed could help to achieve a more satisfactory outcome and to lower the rate of recurrence and other complications. Hopefully, this theory could provide theoretical guidance and support for clinicians to perform better diagnosis and treatment for patients.

## Conclusion

Arthroscopic management with T-shaped capsulotomy of hip SC brought an excellent postoperative outcome with no recurrence, shorter hospitalization and rehabilitation time, and faster return to life. Wedged clump loose bodies manifested distinguishably in terms of radiological appearance, arthroscopic morphology, and surgical strategy.

## Data Availability

All data generated or analyzed during this study are included in the article.
